# Parental Internet Use and Lifestyle Factors as Correlates of Prolonged Screen Time of Children in Japan: Results From the Super Shokuiku School Project

**DOI:** 10.2188/jea.JE20170100

**Published:** 2018-10-05

**Authors:** Masaaki Yamada, Michikazu Sekine, Takashi Tatsuse

**Affiliations:** Department of Epidemiology and Health Policy, Graduate School of Medicine and Pharmaceutical Sciences, University of Toyama, Toyama, Japan

**Keywords:** internet, screen time, parent, Super Shokuiku School Project, food education

## Abstract

**Background:**

Prolonged screen time (ST), which includes TV viewing and gaming on smartphones and computers, is linked to poor health. Our aim was to explore the associations between school children with prolonged ST and parental internet use (IU) and lifestyles in Japan.

**Methods:**

Children aged 6 to 13 years from the Super Shokuiku School Project, were surveyed using questionnaires in 2016. The survey assessed the grade, sex, and lifestyle of 1,659 children and parental internet use (IU) and lifestyle using Breslow’s seven health behaviors. IU consisted of internet surfing and gaming on personal computers (PC), smartphones, or consoles. Three or more hours of ST was defined as prolonged ST, and its correlates were analyzed using logistic regression.

**Results:**

Of all, 643 (38.8%) children spent ≥2 hours/day of ST on a week day, whilst 153 (9.2%) children spent ≥3 hours/day. Prolonged ST was significantly associated with children in higher grade (odds ratio [OR] 1.74; 95% confidence interval [CI], 1.20–2.51), boys (OR 2.16; 95% CI, 1.49–3.14), skipping breakfast (OR 1.88; 95% CI, 1.05–3.35), late bedtime (OR 1.80; 95% CI, 1.15–2.82), physical inactivity (OR 1.79; 95% CI, 1.12–2.87), father’s IU ≥2 hours/day (OR 2.35; 95% CI, 1.52–3.63), mother’s prolonged IU ≥2 hours/day (OR 2.55; 95% CI, 1.43–4.52), mothers with unhealthy behaviors (OR 1.81; 95% CI, 1.05–3.13), no rule setting governing screen time (OR 2.41; 95% CI, 1.63–3.58), and mothers with full-time employment (OR 1.95; 95% CI, 1.06–3.64).

**Conclusions:**

Prolonged ST among Japanese children was strongly associated with parental IU, no set rules for ST, and mother’s unhealthy lifestyles. To reduce children’s ST, parental engagement is warranted in the intervention strategy.

## INTRODUCTION

New information and communication technologies have become common for children and adults in Japan. A 2016 white paper from Japan’s Ministry of Internal Affairs and Communications indicated that the average time of TV viewing for Japanese adults was about 3 hours/day, and internet use was about 1.5 hours/day in 2015.^1^ While the length of time for viewing TV remains unchanged, that of internet use among adult has been increasing steadily (from 71.6 in 2012 to 90.4 minutes in 2015).^1^ Although the use of these media are becoming essential for children and adults, there have been a number of reports on the harmful effects of prolonged screen time (ST) in children,^2^ which includes viewing of videos and TV, gaming on consoles or smartphones, and internet use (IU). In 2004, The Japanese Pediatric Association proposed to parents that ST be reduced in all growing children to nurture wholesome family relationships.^3^

Prolonged ST in children has been associated with unfavorable health and social outcomes, such as obesity,^4^^,^^5^ physical inactivity,^6^ aggressive behaviors,^7^^,^^8^ and poor academic achievement.^9^ Given these health implications, understanding modifiable factors that contribute to reducing ST among children is essential. From previous research, children’s age, parent’s long hours of TV viewing, and no rule setting for ST at home have been found to be clearly associated with prolonged ST among children.^10^^–^^12^ Therefore, a relationship between parents and children was assessed, and family-based interventions were recommended, to decrease ST in children.^13^ However, many reports of ST correlates, which may lead to practical interventions, have not considered the relationship with the general lifestyle of the parents. Moreover, these studies have been conducted in western countries. Research about modifiable correlates of ST of children is insufficient in Japan.

The exponential growth in different types of screen technologies, and IU on smartphones or tablet PCs are increasingly being viewed as detrimental factors for children.^3^ To date, it is well known that parents’ lifestyles associate with those of their children and are crucial risk factors for their children’s health outcomes.^14^^–^^16^ An online survey by the Japanese government about IU in the youth reported that the average time of parental IU was almost 2 hours/day, highlighting that 35.1% of all parents spent 2 or more hours daily on IU.^17^ However, the impact of parental IU on child health or behavior has not been fully surveyed. We hypothesized that parental IU, as well as general lifestyles, might have strong associations with prolonged ST in children. Given the rapid technological development, and increase in the time spent on the internet, more recent data are needed to determine modifiable correlates of prolonged ST.

The purpose of our study was to report children’s ST from a recent survey and to explore correlates of prolonged ST among elementary school children in Japan.

## METHODS

### Participants and the Super Shokuiku School Project

The Super Shokuiku School Project is a food education project which was supported by Japan’s Ministry of Education, Culture, Science and Technology (MEXT).^18^^,^^19^ The overall purposes of the project were to promote healthy lifestyles in school children and improve their health through food education, appreciation of food producers, and participation in farming events. The project was evaluated using repeated questionnaire surveys before and after food education. The baseline survey (Phase 1) was conducted before food education, in May 2014. The follow-up surveys were conducted after food education, in December 2014 (Phase 2) and January 2016 (Phase 3). We obtained detailed information on media use and parental lifestyle factors from phase 3 and conducted a cross-sectional study to assess family relationships and health behaviors. Participants were 2,129 children aged 6 to 13 years who belonged to 5 elementary schools in Takaoka city, Japan. Twenty children were excluded due to illiteracy in Japanese of their parents. A total of 1,987 children agreed to participate in our survey and returned the questionnaires (response rate: 94.2%). The survey was approved by the Ethics Committee of the University of Toyama. Written informed consent was obtained from the participants’ parents, and participation was voluntary.

### Questionnaire

The children and their parents completed questionnaires and returned the questionnaires to their schools. The questionnaires for children queried information on sex, grade, food knowledge, total ST on a week day, other lifestyles, and self-rated health. Questionnaires for parents included assessment of their own lifestyles, time of IU at home on a weekday, perceived family affluence, and parents’ employment status.

### Children’s lifestyle and obesity

Lifestyles (breakfast, physical activity, ST, rules that limit ST with parents, and sleep habits) and health status of children were assessed. Responses to breakfast were coded into two groups as follows: ‘having breakfast every day’ or ‘sometimes to usually skipping breakfast’. Physical activity was divided into three levels: ‘very often’, ‘often’, or ‘rarely to almost never’. We asked the total ST on a weekday, which included TV and DVD viewing; gaming on smartphone, computers, or consoles; and IU. Answers on ST were categorized from 1 to 6, as follows: 1, no or almost no ST; 2, <1 hr; 3, 1 hr to <2 hr; 4, 2 hr to <3 hr; 5, 3 hr to <4 hr; and 6, ≥4 hr. Children were then divided into three groups: ‘<2 hr’, ‘2 hr to <3 hr’, or ‘≥3 hr’. Referring to a report from the Japan Pediatric Association, which recommends a restriction of total ST to within 2 hours a day,^3^ we defined ‘≥3 hr of ST’ as prolonged ST. We also asked whether a rule has been set to limit ST (yes or no). Response to bedtime was categorized into three groups: ‘<10:00 p.m.’, ‘10:00 p.m. to <10:30 p.m.’, or ‘≥10:30 p.m.’. Following reports that the average bedtimes for Japanese elementary school children were 9:44 p.m. for grade 3 and 10:04 p.m. for grade 6,^20^ we defined ‘≥10:00 p.m.’ as late bedtime for children in grade 1 to 3, while ‘≥10:30 p.m.’ was defined as late for grade 4 to 6. The validity of the lifestyle questionnaire that assessed physical activity and sleep was examined in our previous study.^21^ In that study, frequent physical activity was significantly correlated with increased energy expenditure, mean step, and mean activity count per day on the Actiwatch (*P* < 0.05 for a liner trend test). The correlation coefficient between subjective and objective records was 0.97 (*P* < 0.001) for assumed amount of sleep.^22^

We requested children’s height and weight from parents, which were measured in January 2016 by trained school nurses at their schools. The health screen is performed on an annual basis in April, September, and January according to the School Health Law. The percentage of obesity is usually assessed in the School Health Statistics Research in Japan.^23^ Age-, sex-, and height-based ideal body weight (BW; kg) was calculated based on the data from the national statistics,^23^ from which the percentage of deviance from the ideal BW was calculated ((individual BW − ideal BW)/ideal BW). The percentage of obesity was reported to have strong correlation with BMI Z score (*R*^2^ = 0.957 for boys and 0.949 for girls).^24^ Children who are more than 20 percentage over their ideal BW are regarded as obese in Japan, which Isoshima et al reported to be almost equivalent to those above the 95th percentile of their age- and sex-specific BMI.^24^

### Parental lifestyle, employment status, family affluence and internet use

To assess general parental lifestyles, Breslow’s seven good health-related behaviors^25^ were used. These comprise seven indicators of good health: (1) adequate sleep time, (2) no smoking, (3) appropriate weight control, (4) not drinking excessively, (5) regular physical activity, (6) no skipping of breakfast, and (7) not frequently snacking. Breslow’s seven good health-related behaviors are widely accepted in western countries.^25^ The higher the number of healthy behaviors practiced, the lower the morbidity; additionally, mortality rates are lower for people who practice the seven health behaviors. In our study, the total number of behaviors out of the seven showed a normal distribution among parents. Therefore, we divided parents into three groups based on the number of behaviors: low (0–3), middle (4–5), and high (6–7). The higher the number, the healthier the participant. Parental employment status was categorized into three groups: full-time, part time, or unemployed. Regarding socioeconomic status (SES), we asked parents about their perceived family affluence. The responses to the question were divided into three categories: ‘affluent’, ‘neither’, or ‘not affluent’. Finally, we asked the total time for parental IU at home on a weekday. The time for parental IU was divided into two categories of ‘<2 hr’ or ‘≥2 hr’ based on the report that the average time spent on the internet by Japanese adults in their thirties was about one and a half hours in 2015.^1^

### Statistical analysis

Taking children’s grade (age) difference for lifestyles and ST into consideration, we divided children into two groups, lower (1–3) and higher (4–6) grades, for descriptive data. Chi-squared test was performed to compare variables by groups. Then, to assess the between-school (cluster) variance in children’s ST, interclass correlation (ICC) was calculated with the six categories of ST defined as continuous variables. Finally, univariate and multivariate logistic regression analyses were conducted to evaluate the strength of the associations of the lifestyle of children and parents with prolonged ST, using the forced entry methods. Father’s employment status was excluded from the regression analysis because almost all the fathers were full-time workers (99.5%). The odds ratios (OR) and 95% confidence intervals (CI) were calculated. Analyses were conducted using the statistical package for social scientists (SPSS) version 22.0 J (SPSS, Chicago, IL, USA). A two-tailed *P*-value of less than 0.05 was considered statistically significant.

## RESULTS

Out of 1,987 who returned their questionnaires, 1,659 school children (78.7%; 828 boys and 831 girls) who answered all the questionnaire items relevant to the present study were included in our analyses. About 40% of children (*n* = 643) spent ≥2 hr/day on ST, and 9.2% (*n* = 153) spent ≥3 hr/day. There was no significant difference in sex, obesity, parental IU, numbers of health behaviors, or perceived family affluence between children in lower (1–3) and higher (4–6) grades. However, children in higher grades were more likely to skip breakfast, go to bed late, and have prolonged ST, while children in lower grades were more likely to be physically active and have unemployed mothers (Table 1). ICC in children’s ST was only 4.6%.

**Table 1.** Characteristics of children by grade (*n* = 1,659)

		Low (1st–3rd) *n* = 824		High (4th–6th) *n* = 835		

Age, years, mean (SD)		7.9 (0.87)		11.0 (0.85)		*P* value
		*n*	%	*n*	%	
Sex	Boy	414	50.2	414	49.5	0.806

Breakfast	Skipping	36	4.4	71	8.5	<0.001

Bedtime, p.m.	<10	732	88.8	448	53.7	<0.001
	10≤ to <10:30	73	8.9	262	31.3	
	≥10:30	19	2.3	125	15.0	

Physical activity	Very often	233	28.3	238	28.5	<0.001
	Often	403	48.9	332	39.7	
	Rarely or almost never	188	22.8	265	31.8	

Obesity	Obese	67	8.1	64	7.6	0.715

Father’s IU at home, h/day	≥2	123	14.9	116	13.9	0.576

Mother’s IU at home, h/day	≥2	45	5.5	48	5.8	0.831

Father’s health behaviors in Breslow’s	High (6–7)	196	23.8	163	19.5	0.080
	Middle (4–5)	337	40.9	375	45.0	
	Low (0–3)	291	35.5	297	35.5	

Mother’s health behavior in Breslow’s	High (6–7)	235	28.5	215	25.7	0.129
	Middle (4–5)	445	54.0	458	354.9	
	Low (0–3)	144	17.5	162	19.4	

Rule setting to restrict ST	No	137	16.6	161	19.3	0.160

Father’s employment status	Fulltime	821	99.6	829	99.3	—
	Part time	1	0.1	2	0.2	
	Unemployed	2	0.2	4	0.5	

Mother’s employment status	Fulltime	326	39.6	344	41.2	0.010
	Part time	355	43.1	389	46.6	
	Unemployed	143	17.4	102	12.1	

Perceived Family Affluence	Affluent	233	28.3	224	26.9	0.793
	Neither	391	47.5	400	48.0	
	No affluent	200	24.3	211	25.2	

Children’s ST, h/day	<2	542	65.8	474	56.8	<0.001
	2≤ to <3	229	27.8	261	31.2	
	≥3	53	6.4	100	12.0	

IU, Internet use; ST, screen time.

*P* values in Pearson’s chi-square test were shown.

In Table 2, logistic regression analyses were performed to determine the strength of association of the lifestyle of children and parents with prolonged ST in children. For univariate analysis, children in higher grades (OR 1.98; 95% CI, 1.40–2.18), being a boy (OR 1.78; 95% CI, 1.27–2.51), skipping breakfast (OR 2.63; 95% CI, 1.58–4.37), late bed time (OR 2.45; 95% CI, 1.64–3.65), physical inactivity (OR 2.15; 95% CI, 1.39–3.33), both father’s and mother’s IU for ≥2 hr/day (OR 2.92; 95% CI, 2.00–4.26 for father’s and OR 4.39; 95% CI, 2.70–7.16 for mother’s), mothers with low Breslow’s behaviors (OR 2.99; 95% CI, 1.87–4.80), no rule setting to limit ST at home (OR 2.64; 95% CI, 1.84–3.78), mothers with full-time employment status (OR 1.87; 95% CI, 1.05–3.32), and no family affluence (OR 1.62; 95% CI, 1.02–2.59) were associated with prolonged ST in children. In multivariate analysis, children in higher grades (OR 1.74; 95% CI, 1.20–2.51), being a boy, (OR 2.16; 95% CI, 1.49–3.14), skipping breakfast (OR 1.88; 95% CI, 1.05–3.35), late bed time (OR 1.80; 95% CI, 1.15–2.82), physical inactivity (OR 1.79; 95% CI, 1.12–2.87), both father’s and mother’s IU for ≥2 hr/day (OR 2.35; 95% CI, 1.52–3.63 for father’s and OR 2.55; 95% CI, 1.43–4.52 for mother’s), mothers with low Breslow’s behaviors (OR 1.81; 95% CI, 1.05–3.13), no rule setting to limit ST at home (OR 2.41; 95% CI, 1.63–3.58), and mother with full-time employment status (OR 1.96; 95% CI, 1.06–3.64) were significantly associated with prolonged ST. The association between family affluence and prolonged ST seen in univariate analysis was not observed in the multivariate analysis.

**Table 2.** Logistic regression analysis on children’s prolonged ST (*n* = 1,659)

		ST 3 h/day or more (%)	univariate		multivariate	
	
			OR	95% CI	OR	95% CI
Grade	**High (4–6th)** (/Low)	**12.0/6.4**	1.98^***^	**1.40–2.81**	1.74^**^	**1.20–2.51**

Sex	**Boy** (/Girl)	**11.4/6.9**	1.78^***^	**1.27–2.51**	2.16^***^	**1.49–3.14**

Breakfast	**Skipping** (/No Skip)	**19.6/8.5**	2.63^***^	**1.58–4.37**	1.88^*^	**1.05–3.35**

Bedtime, p.m.	**Later bed time** (/Low grader for <10, High grader for <10:30)	**17.5/8.0**	2.45^***^	**1.64–3.65**	1.80^**^	**1.15–2.82**

Physical activity	Very often	7.2	1		1	
	Often	7.4	1.02	0.65–1.59	0.94	0.59–1.50
	**Rarely or almost never**	**14.3**	2.15^***^	**1.39–3.33**	1.79^**^	**1.12–2.87**

Obesity	Obese (/Non-Obese)	13.1/8.9	1.54	0.90–2.64	1.32	0.72–2.42

Father’s IU at home, h/day	**≥2** (/<2)	**19.2/7.5**	2.92^***^	**2.00–4.26**	2.35^***^	**1.52–3.63**

Mother’s IU at home, h/day	**≥2** (/<2)	**28.0/8.1**	4.39^***^	**2.70–7.16**	2.55^***^	**1.43–4.52**

Father’s health behaviors in Breslow’s	High (6–7)	8.4	1		1	
	Middle (4–5)	7.6	0.90	0.57–1.43	0.77	0.47–1.28
	Low (0–3)	11.8	1.46	0.93–2.29	0.94	0.56–1.59

Mother’s health behavior in Breslow’s	High (6–7)	6.7	1		1	
	Middle (4–5)	7.6	1.16	0.74–1.80	0.90	0.55–1.45
	**Low (0–3)**	**17.6**	2.99^***^	**1.87–4.80**	1.81^*^	**1.05–3.13**

Rule setting to restrict ST	**No** (/Yes)	**17.4/7.4**	2.64^***^	**1.84–3.78**	2.41^***^	**1.63–3.58**

Mother’s employment status	**Full time**	**10.9**	1.87^*^	**1.05–3.32**	1.96^*^	**1.06–3.64**
	Part time	8.7	1.46	0.82–2.61	1.47	0.79–2.75
	Unemployed	6.1	1		1	

Perceived Family Affluence	Affluent	7.2	1		1	
	Neither	9.4	1.33	0.87–2.03	1.08	0.68–1.71
	**No affluent**	**11.2**	1.62^*^	**1.02–2.59**	1.07	0.64–1.79

CI, confidence interval; IU, internet use; OR, odds ratio; ST, screen time.

^*^: *P* < 0.05, ^**^: *P* < 0.01, ^***^: *P* < 0.001.

Table 3 shows the association between parental IU and each health behavior, and children’s prolonged ST. Improper weight control, no regular exercise and frequent snacking were behaviors of the fathers found to be associated with prolonged ST in univariate analysis, however, these associations were not observed in the multivariate analysis. On the other hand, mother’s behaviors, except frequent snacking, were associated with prolonged ST in the univariate analysis, whilst the significant associations of inadequate sleep (OR 1.45; 95% CI, 1.01–2.07) and excessive drinking (OR 2.30; 95% CI, 1.32–4.01) was observed in the multivariate analysis. Both parental IU were strongly associated with children’s prolonged ST (OR 1.22; 95% CI, 0.79–1.90 for 1 to <2 hr/day and OR 2.29; 95% CI, 1.45–3.60 for ≥2 hr/day in father’s IU; OR 1.54; 95% CI, 0.99–2.38 for 1 to <2 hr/day and OR 3.51; 95% CI, 1.99–6.20 for ≥2 hr/day in mother’s IU).

**Table 3.** Associations of parental IU and lifestyles with children’s prolonged ST (*n* = 1,659)

		ST 3 h/day or more (%)	univariate		multivariate	
	
			OR	95% CI	OR	95% CI
Father’s IU at home, h/day	<1	6.7	1		1	
	1 ∼ <2	9.6	1.48	0.98–2.24	1.22	0.79–1.90
	**≥2**	**19.2**	3.32^***^	**2.21–4.97**	2.29^***^	**1.45–3.60**

Mother’s IU at home, h/day	<1	6.9	1		1	
	**1 ∼ <2**	**13.1**	2.02^**^	**1.35–3.01**	1.54	0.99–2.38
	**≥2**	**28.0**	5.12^***^	**3.15–8.59**	3.51^***^	**1.99–6.20**

Father’s health behaviors in Breslow’s (not good/good behavior)	Adequate sleep (no/yes)	9.1/9.3	0.98	0.70–1.37	0.75	0.52–1.08
	Smoking (yes/no)	10.8/8.0	1.39	0.99–1.94	1.16	0.80–1.69
	**Proper weight control (no/yes)**	**11.2/8.0**	1.46^*^	**1.04–2.04**	1.07	0.74–1.54
	Excessive drinking (yes/no)	10.7/8.8	1.24	0.85–1.82	0.94	0.62–1.42
	**Regular exercise (no/yes)**	**10.3/6.9**	1.56^*^	**1.06–2.30**	1.35	0.82–2.05
	Skipping breakfast (yes/no)	10.8/8.6	1.27	0.89–1.83	0.96	0.64–1.43
	**Snacking often (yes/no)**	**11.2/7.9**	1.48^*^	**1.06–2.07**	1.02	0.70–1.50

Mother’s health behaviors in Breslow’s (not good/good behavior)	**Adequate sleep (no/yes)**	**11.5/7.4**	1.62^**^	**1.16–2.27**	1.45^*^	**1.01–2.07**
	**Smoking (yes/no)**	**15.4/8.4**	1.98^**^	**1.27–3.08**	1.41	0.84–2.35
	**Proper weight control (no/yes)**	**12.3/7.9**	1.65^*^	**1.17–2.32**	1.04	0.71–1.52
	**Excessive drinking (yes/no)**	**20.4/8.4**	2.78^***^	**1.66–4.63**	2.30^**^	**1.32–4.01**
	**Regular exercise (no/yes)**	**10.1/5.9**	1.79^*^	**1.11–2.89**	1.57	0.96–2.55
	**Skipping breakfast (yes/no)**	**13.5/8.5**	1.72^*^	**1.12–2.64**	1.17	0.72–1.89
	Snacking often (yes/no)	9.8/8.5	1.17	0.84–1.64	0.92	0.64–1.32

CI, confidence interval; IU, internet use; OR, odds ratio; ST, screen time.

^*^: *P* < 0.05, ^**^: *P* < 0.01, ^***^: *P* < 0.001.

Mother’s employment status and perceived family affluent were adjusted in multivariate analysis.

## DISCUSSION

Our results showed that about 10% of children spent 3 or more hours of total ST on a weekday, which was less prevalent than in the national survey reporting that 34.0% of children spent 3 or more hours on TV and DVD viewing,^26^ and that parental IU and rule setting at home were strongly associated with children’s ST. These associations were stronger than that of children’s sex, age, and lifestyle. Moreover, we observed an association between mother’s lifestyle and children’s ST. These findings could help health practitioners and media researchers who work with children to understand modifiable factors that contribute to reducing ST among children.

### Internet use

Previous literature has shown a significant relationship between screen-based behaviors of both parents and children,^10^^–^^12^^,^^27^ much of which focused mainly on TV viewing. We used parental IU instead of TV viewing because of the following three reasons: First, IU requires much more attention of users than TV viewing does. Second, the 2016 white paper from Japan’s Ministry of Internal Affairs and Communications reported that the average time of TV viewing among those aged in their 30s was decreasing year by year, while their average time spent on IU was steadily increasing.^1^ Third, it seems difficult to co-view smart media and communicate with other family members because the screen size is usually smaller than that of TV. Moreover, parental IU is especially thought to be a detrimental factor in Japan’s modern society,^3^ where multiple devices for IU, including the smart phone, have become dramatically more common in recent years. The percentage of people owning a smart phone among Japanese was 9.7% in 2002, a rate which soared up to 72.0% in 2015. Nowadays, 83.0% of Japanese are reported to connect to the internet.^1^ For these reasons, we hypothesized that parental IU would have strong impact on their children’s ST. To the best of our knowledge, this is the first research to explore the association between parental IU and children’s ST in Japan. Both mother’s and father’s parental IU were strongly associated with prolonged ST in children, even after adjusting for children’s lifestyle factors. A plausible explanation of the association may be modeling effect, rather than co-viewing. Parents who use internet longer do not give children another model of how to spend their leisure time and may displace the time that children would otherwise spend engaged in emotional communications with their parents. In fact, children of parents who spent 2 or more hours on IU reported less communication in their family than that of parents spending less than 2 hours (data are not shown). We should treat parental IU as a critical factor for reducing children’s ST.

### Rule setting

In our study, an existence of rule to limit children’s ST was found to be significantly associated with fewer reports of prolonged ST. This corresponds with other research.^10^^–^^12^^,^^28^ We found that the majority of children (82.0%) had a rule with their parents to restrict total ST. This percentage was higher than other previous reports, such as 60.9% in the United States,^27^ 36.1% in the Czech Republic and Slovakia,^12^ and about 50% in China.^11^ Japanese health practitioners should continuously recommend more families to apply this kind of restriction at home.

### Basic characteristics, lifestyle, and obesity

Other studies have shown that being a boy, an older age, physical inactivity, unhealthy dietary behaviors, sleep problems, and obesity were associated with prolonged ST in children.^10^^,^^11^^,^^29^^–^^32^ In line with these results, we found significant associations between prolonged ST and being a boy, children in higher grades, physical inactivity, skipping breakfast, and late bed time. Although several previous reports have shown obesity to be associated with ST,^29^^,^^33^^–^^35^ there was no significant relationship between prolonged ST and obesity in our study. This may be due to the much lower prevalence of obesity in our study (7.8%) compared with others; prevalence of obesity was 23.7% in Irish and 53.8% in Iranian children in other studies.^34^^,^^35^

Mother’s unhealthy lifestyle, which was assessed using the Breslow’s seven health-related behaviors, was associated with prolonged ST in children. From the specific analysis of parental lifestyle, children of mothers who did not have adequate sleep, smoked, drank excessively, and did not exercise regularly were more likely to have prolonged ST. On the other hand, father’s health behaviors were not associated with prolonged ST in children. Our results were in line with the previous literature from Sekine et al.^15^ They reported that children’s unhealthy lifestyles, such as skipping breakfast, inadequate physical activity, and prolonged TV viewing, were associated with mother’s obesity but not with father’s obesity. A plausible explanation about the difference may be that mothers have played a key role in rearing children in Japan. This is one of the few limited studies to clarify the relationship between parental lifestyle and children’s prolonged ST. It is important for health providers and guardians of children to consider mother’s lifestyle, as well as children’s own lifestyle, in the reduction of ST in children.

### SES and parental employment status

The association between SES and ST in children remains unclear,^10^ although negative associations have been observed in developed countries (children in higher SES had less ST),^31^^,^^36^ while positive associations have been observed in developing countries.^35^ In our study, we found a significant relationship in the univariate analysis, which was not observed in the multivariate model. This difference may have resulted from the method of analyses. Previous researches, which were conducted in the United Kingdom and Australia, did not include parental and children’s lifestyle and media.^31^^,^^36^ Thus, these lacking factors may have a strong influence on children’s ST, rather than SES.

We also found a significant relationship with mother’s employment status. Compared to mothers who are unemployed or with part-time jobs, those with full-time employment were likely to let their children have long ST. Our results supported previous studies that showed association between unhealthy children’s lifestyle and mother’s longer work schedule.^15^^,^^37^ Magee et al showed that children of mothers working ≥45 hr/week had later bed time and higher OR of short sleep hours. Sekine et al pointed out that children of mothers working full-time were more likely to watch TV for longer hours, had frequent snacks, and went to bed late.^15^ In our study, children of mothers working part time did not show a significant increase in ST compared to children with unemployed mothers. Duration of hours of work of parents may influence children’s lifestyle. However, we cannot insist that mothers should work less in the current society in Japan, where a growing number of mothers are full-time workers. This may be impossible for many families and could have adverse influences, such as loss of income; however, what counts is to recognize the children’s lifestyle activity and lessen any negative effect on children, by providing playgrounds, daycare centers with physically active games, and tutoring from sufficient staff or health practitioners.

### Study strengths and limitations

To the best of our knowledge, this is the first study to examine the strength of associations of prolonged ST in school children with their lifestyle, as well as parental IU and lifestyle, in Japan. We found that both parents IU and rule setting had stronger relationships with prolonged ST in children than children’s lifestyle did. Thus, interventions for reducing ST among children must involve parents as well as children. Guardians and childcare staff should be educated on the importance of limiting length of time of parental IU, and of rule setting in reducing ST in children.

Our study has some limitations. First, our study design was cross-sectional, so we were not able to infer causation. However, reverse causality, in which children’s prolonged ST led to longer parental IU, is unlikely. Limiting parental IU may be a plausible intervention for prolonged ST in children. Second, questionnaires could not reflect the actual lifestyle and anthropometry data. Children might report their lifestyles better than they actually led according to social expectation. This kind of bias could occur among parents as well. The national data conducted in 2014 reported that about 35% of parents of elementary school children spent 2 or more hours in IU a day.^17^ Our questionnaire might not reflect the actual time duration. Objective date will be needed in future study, especially about time, such as ST and IU. Third, our questionnaire asked only the total ST for children and IU for their parents. To focus on recreational use, time spent on studying for children or working for parents should have been divided. Fourth, our study did not include potential factors, such as number of TVs in a household, the existence of a TV, game or other media in child’s bedroom, parental TV viewing, and children’s characteristics, such as attentional abilities and self-regulation behaviors.^38^^,^^39^ Future studies are needed to test the association or effect of these factors. Finally, our research restricted the participants to dual-parent families to assess both parent’s IU and lifestyle. We could not explore single-parent families. Given our results, children of a single mother working longer may have longer ST than in a dual-parent family. Further studies that include single-parent families are also needed for child health promotion.

### Conclusions

The present study provides data about correlates of prolonged ST among Japanese elementary school children. Parental IU and rule setting have stronger relationships with prolonged ST in children than children’s own lifestyle, such as skipping breakfast, late bedtime, and physical inactivity. In addition, mother’s unhealthy lifestyle correlates strongly with prolonged ST in children. In Japan, where the social trend of people owning and spending time on smartphones or tablet PC is increasing, health practitioners and guardians of children should be informed about these results. The engagement of parents in creating a healthier family environment is warranted as an intervention strategy for reducing ST in children.
